# Albert Piette and lived (non-)religion: Conceptual and methodological considerations

**DOI:** 10.1177/00084298241236946

**Published:** 2024-03-17

**Authors:** Lori G Beaman, Lauren Strumos

**Affiliations:** Department of Classics and Religious Studies, University of Ottawa, Canada; Department of Classics and Religious Studies, University of Ottawa, Canada

**Keywords:** Lived religion, non-religion, social sciences of religion, qualitative research, community gardens, Religion vécue, non-religion, sciences sociales de la religion, recherche qualitative, jardins communautaires

## Abstract

This article considers how the work of Albert Piette can enhance a sociological understanding of religion as lived in everyday life. Although Piette is critical of sociologists in *La Religion de près* (1999), his work resonates with scholarship on ‘lived religion’ by contemporary sociologists of religion. This article locates points of overlap and divergence between Piette and scholars of lived religion, and then turns its attention towards the emergent study of lived non-religion. It concludes that while Piette’s micro-level approach to religious life has limitations for sociologists, he still offers conceptual and methodological perspectives that contribute to research on lived religion and non-religion. To demonstrate this argument, Piette’s work is drawn into a discussion of the Nonreligion in a Complex Future’s Community Gardens research project, which explores how religious and non-religious actors enact and construct their (non-)religion in community gardens.

## Résumé

Cet article examine comment les travaux d’Albert Piette peuvent améliorer la compréhension sociologique de la religion telle qu’elle est vécue dans la vie quotidienne. Bien que Piette critique les sociologues dans *La Religion de près* (1999), son travail entre en résonance avec les travaux sur la “religion vécue” menés par les sociologues de la religion contemporains. Cet article identifie les points de chevauchement et de divergence entre Piette et les spécialistes de la religion vécue, puis s’intéresse à l’étude émergente de la non-religion vécue. Il conclut que si l’approche micro-niveau de la vie religieuse de Piette présente des limites pour les sociologues, elle offre néanmoins des perspectives conceptuelles et méthodologiques qui contribuent à la recherche sur la religion vécue et la non-religion. Pour démontrer cet argument, le travail de Piette est intégré dans une discussion sur le projet de recherche Nonreligion in a Complex Future’s Community Gardens, qui explore la manière dont les acteurs religieux et non religieux mettent en œuvre et construisent leur (non-)religion dans les jardins communautaires.

## Introduction

It is unfortunate that the work of Albert Piette is little known amongst English-speaking sociologists and social scientists of religion. In 1999, *La Religion de près* was published, in French, at a time when sociology of religion was preoccupied with developing theories of secularization. The notion that religious decline and privatization characterize modernity became a core theme to be elaborated and refuted. The relationship between religion and modern societies occupied much of the scholarly landscape (Beyer, 1990, 1994; Casanova, 1994; Martin, 1996; Warner, 1993). These discussions frequently overshadowed empirical research focused on individual and group practices, continued religious participation, and a deeper understanding of religious disaffiliation and reconstitution. In particular, scholars in sociology, anthropology and religious studies were refining their approach to religion, moving beyond institutional engagement and the focus on congregations to consider the constitution of everyday practices as religion. This line of scholarship, which has come to be known as ‘lived religion’ in the social sciences of religion, is congruent with Piette’s methodology for capturing ‘religion in the making’.

This article situates Piette’s work in relation to the scholarship on lived religion. It considers what *La Religion de près* can contribute to our understanding of religion and how it is constituted and lived in everyday life. However, we are concerned not only with religion and everyday life, but also with the construction of everyday non-religion. Specifically, we are interested in the complex ways that social actors construct, deconstruct and indeed are indifferent to religion in their everyday lives. Research on non-religion has exploded during the past decade or so, fuelled in part by the dramatic decrease in participation in organized religion, especially in countries that have traditionally been dominated by Christianity. In this article, we bracket the secular, secularity and secularization, and think instead about the complex ways that social actors enact and construct (non-)religion. It is here that Piette’s work is especially helpful. We consider what may be gained by greater attention to his approach by reflecting on a research project on community gardens that is currently underway. Our reflections focus on his methodological impact as well as his embracing of the contradictions and complexities that emerge from everyday life.

## A sociological reading of Piette

In the introductory chapter of *La Religion de près*, Piette (1999: 23) criticizes sociologists of religion for subordinating religion to ‘the external authority of sociology’ (‘l’autorité extérieure du sociologie’). He notes their overattachment to secularization and modernity as interpretative frameworks, and essentialist definitions that reflect biased notions of what counts as ‘religion’.^1^ Piette aims to displace these approaches with a more experiential one grounded in religious practice. Describing the contents of *La Religion de près*, Piette writes:

Nous décrirons donc ce que font les gens quand ils s’occupent d’une religion, quand ils font du religieux, dans la confusion et l’émiettement quotidien des rencontres. Nous plongerons fait au cœur de l’activité religieuse en suivant le plus près possible les acteurs, dans leurs interactions locales, les détails anecdotiques des échanges verbaux et les gestes les plus insignifiants. (Piette, 1999: 33–34)^2^

Piette takes his reader on an ‘ethnographic journey’ that closely follows a priest and Catholic laity in a French diocese. He brackets defining religion and is instead guided by people’s actions and conversations, arguing that doing so is necessary to take religious activity seriously (Piette, 1999: 29–30). This bottom-up approach, according to Piette, distinguishes his work from the sociologists of religion who propose macro-level definitions or explanations of religion. It further highlights the diversity and fluidity of ordinary religious life. Religion, for Piette, is not a fixed or abstract object of study but something that unfolds through concrete, localized activity in the everyday. In other words, religion is not ‘made’ but always ‘in the process of being made’ (‘en train de se fabriquer’; Piette, 1999: 32).

In considering what distinguishes Piette’s approach, we turn to James Beckford’s assessment of his contribution. Beckford highlights the ‘freeze frame’ technique used by Piette, which is essentially a detailed inspection of the minutia of social life. Beckford (2017: 183) points out that Piette is less interested in ‘revealing the coherence or functionality of social processes or typical situations than with showing the indeterminacy of acts and gestures that might always develop in paradoxical or unexpected ways’. Such close attention, for Piette, should be at the core of the social scientific study of religion, rather than the imposition of a conceptual or theoretical framework. This method is particularly evident in *La Religion de près* when he criticizes the destroying of the objective of inquiry through a ‘fracture hétéro-explicatrice’ (‘Hetero-explanatory fracture’). Piette’s dislike of the scholarly imposition of concepts and interpretive interventions is also present in his later writings, such as that found in *Anthropology and the Existential Turn*, in which he states:

In the social sciences of religion, the temptation to overinterpret jeopardizes the description and analysis of beliefs, or more specifically modes of believing. Religious beliefs are often treated as synonymous with homogeneous, shared cultural representations, as if adherence, acceptance, and the mode of belief were self-evident. (Piette, 2015: 191)

Pausing here to reflect for a moment, we think that the paradoxes that necessarily arise from consideration of everyday life are frequently lost in a too-quick move to interpretation or a preconceived theoretical framework. It is these paradoxes that are at the heart of (non-)religion – a point we will return to later in the article.

Beckford also notes Piette’s critique of the sociological tendency to fixate on the new, exciting and deviant. This means that phenomena that are not representative of social life tend to be over-represented and are ‘far removed from the banal realities of ordinary religion’ (Beckford, 2017: 185). These banal realities might be the ‘pearls of social life’ that Les Back (2007: 17) argues are often missed by sociologists, stating pointedly that ‘the texture of the very lives we seek to render is flattened and glossed’. Religion outside of religious institutions and organizations is of interest to Piette as ‘religion in a minor key rather than as a separate field of heightened emotion, of special power or of great symbolic or ritual intensity’ (Beckford, 2017: 186). Beckford’s observation that Piette reveals the interweaving of religion and non-religion, and that he undermines the notions of both the religious field (Bourdieu, 1991) and secularization, is salient to our project. There is, in our minds, a fascinating parallel between Matthew Wood’s (2018) insistence that church committees are an important site of sociological inquiry and Piette’s studies of church committees. This focus on social actors is at the forefront of the study of religion for both researchers.

Although Piette is critical of sociologists of religion for ignoring the banalities of ordinary religion, his work overlaps with sociological research on lived religion. Scholars who study lived religion are attuned to the nuance, complexity and fluidity of religion as it is practised in everyday life. They are not concerned with authoritative religious beliefs or teachings, but with how religion is understood, experienced and expressed – or ‘lived’ – by people outside of institutional religious settings. This includes how aspects of religious identity interact with other areas of life, such as sex and gender (Beyer and Ramji, 2013; McGuire, 2008; Neitz, 2014). Nancy Ammerman (2020: 9–10) notes that the term ‘lived religion’ was first used by a group of historians and sociologists whose work appeared in the collection *Lived Religion in America*, edited by David Hall and published in 1997, two years before Piette’s *La Religion de près*. This work represents the efforts of social scientists in the mid 1990s to find new ways of understanding the relationship between religion and society outside of secularization and rational-choice theory (Ammerman, 2007, 2020).^3^ In this sense, the lived religion approach and that of Piette find similarity in their discontent with un-nuanced approaches to secularization and other ‘macrosociological interpretations’ of religion.^4^ They are both concerned with capturing the everyday religious practice of individuals.

Scholars of lived religion are attuned to the complexity of religious practice. As Meredith McGuire (2008: 4) indicates, religion at the ‘individual level’ is ‘ever-changing, multifaceted, often messy’ and ‘even contradictory’. Contradictions are the focus of attention by Piette in his description of parish meetings in *La Religion de près*. He describes, for instance, disagreement among the laity over what makes a proper funeral. For some, the presence of the priest and Eucharist is crucial, while others consider this superstitious and emphasize the involvement of the family or community (Piette, 1999: 118). Piette also pays attention to points of tension among those who are dissatisfied with certain aspects of the parish. For example, there are lay members who enjoy the traditional hierarchical structuring of the Catholic Church and then there are those who favour a more democratic arrangement (Piette, 1999: 95). Piette documents such inconsistencies not by concentrating on the ‘official’ views or practices of Catholicism, but by exploring how religious activity, at the level of individual lay members, is understood and expressed through ordinary interactions.

One difference between Piette in *La Religion de près* and sociologists researching lived religion is that the latter concentrate on non-elites and non-institutional contexts (Ammerman, 2016, 2020; McGuire, 2008). Courtney Bender (2003) conducted ethnographic research as a volunteer in a New York City kitchen that prepares and delivers meals to people with AIDS. This approach allowed her to study the ways people talk about religion outside of religious settings, with Bender (2003: 8) concluding that religious language ‘is not bracketed or given special status but rather is part of a dialogue among other practices in particular situations’. Piette is also concerned with detailing the dialogue of actors through ethnography, but he largely limits this interest to an explicitly Catholic context by following the activities of a priest. Although the study of lived religion does not necessitate the exclusion of institutional religion and clergy (Ammerman, 2016; Grigore-Dovlete, 2020),^5^ it largely focuses on religion outside of clearly demarcated religious spaces and on the religious practice of non-elites. Likewise, while Piette’s focus on the priest in *La Religion de près* contradicts the focus of much lived religion scholarship, his approach is not limited to religious institutions and elites (Beckford, 2017).

Crucial to the work of Piette and that of lived religion scholars is the centrality of the voices of social actors. Robert Orsi (2005: 18) highlights this when he reflects on the concept of ‘belief’ in the study of religion: ‘But belief has always struck me as the wrong question, especially when it is offered as a diagnostic for determining the realness of the gods’. Reflecting on this ‘realness’, Orsi (2005: 18) continues: ‘The saints, gods, demons, ancestors, and so on are real in experience and practice, in relationships between heaven and earth, in the circumstances of people’s lives and histories, and in the stories people tell about them’. Through historical and ethnographic research, Orsi provides critical insight into the relationships American Catholics form with divine figures like Saint Jude and the Virgin Mary. He finds that they are subjective and complex, ranging across love, healing, fear, vulnerability and denial. These relationships are not a belief but very real for those Orsi studies, and the depth at which he aims to understand them reflects this. Further, the rich stories Orsi tells bring religion to life, displacing the institution as the core locus of its making to the micro-processes of everyday engagement with saints and priests. In this way, he and Piette are engaged in a similar project. But Orsi’s work is thick with interpretive analysis in a way that Piette’s is not. We might think of Piette’s approach as producing a highly pixilated picture with a singular focus and Orsi’s as a lower-resolution photograph of a broader expanse.

In *La Religion de près*, Piette considers how God, as an invisible entity, is nevertheless present to the parishioners through different modalities. God’s presence can arise through mediations, such as prayer and song, or his presence at events may be detectible through signs (Piette, 1999: 92, 182). In his observations of his research subjects’ approach to God, Piette (1999: 78) concludes that they see God as ‘a hybrid’: he is both ‘present and absent, manufactured and autonomous’. Such autonomy derives from God’s ability to incite the activities of his parishioners; he is an invisible yet influential force in their daily religious lives.^6^ This observation does not assert whether God exists. Doing so falls outside the scope of the social sciences of religion (Beckford, 2003). Instead, Piette is discerning of ‘God’s hybridity’ because he does not diminish or dismiss the place of God as expressed by those he observes. He thus demonstrates how taking seriously the views of other people can offer detailed understandings of ordinary religious practice, as observed through ethnographic research. This is not ‘religious sociology’, as critiqued by Véronique Altglas and Matthew Wood (2018), but rather sociology of religion that observes, listens and describes.

## Moving from religion to non-religion

Having situated Piette’s work in some of the broader sociology of religion literature, we shift our attention to non-religion. While it remains a contested term, ‘non-religion’ is often used by sociologists to indicate a phenomenon that is somehow related to religion while also remaining distinct from religion. Lois Lee (2015: 32) sees this connection as being key to defining non-religion, in that non-religion is ‘not the absence of something (religion) but the presence of something (else), characterized, at least in the first place, by its relation to religion but nevertheless distinct from it’. Johannes Quack (2014) proposes a relational theory of non-religion by drawing on Pierre Bourdieu’s concept of the ‘field’. Quack argues that non-religion should be conceptualized as a phenomenon that is outside yet related to a religious field, whether directly or indirectly. Atheist groups who overtly criticize religion, for example, distinguish themselves from the religious field while being positioned in relation to it through their criticism (Quack, 2014: 451). In this relational approach, non-religion is not static but rather occurs in various relationships with religion that change over time and in different social contexts.

In addition to studying non-religion as a social phenomenon, sociologists of religion have turned their attention to the increase in the number of individuals who identify as having no religion. This group of people, who fall under the umbrella term ‘non-religious’ or ‘nones’, consists of self-identifying atheists, humanists, agnostics, sceptics and spiritual but not religious, and those who are indifferent to religion. Much like those who identify as religious, non-religious identities are heterogenous and shaped by individual experiences, relationships, cultures and sociopolitical contexts (Quack, 2014; Quack et al., 2020; Zuckerman et al., 2016). The lived (non-)religion approach in particular has enabled sociologists of religion to capture the diversity of lived experiences and practices among the non-religious (Beyer, 2015; Salonen, 2018; Zuckerman et al., 2016).^7^ Moreover, being non-religious does not necessarily entail an absence of beliefs and practices typically considered religious. Some non-religious people, for example, believe in an afterlife or higher power and attend religious services (Smith and Cragun, 2019; Thiessen and Wilkins-Laflamme, 2020; Woodhead, 2017). Non-religion as a positive identity, perhaps conceptualized as meaning constructed without reference to religion, has also become an important area of study (Brown, 2017; Cragun and McCaffree, 2021; Lee, 2015; Manning, 2015; Quack et al., 2020; Wilkins-Laflamme, 2022). In other words, non-religion is more than a lack of religion, and this positive or substantive aspect of non-religion as found in the messy world of everyday life is of most interest to us.

The Nonreligion in a Complex Future project is a Canadian-based research project that focuses on identifying the social impact of the rapid and dramatic increase of non-religion. International and comparative, our research sites include Canada as the focal point, with co-investigators, collaborators, advisors and partners in Australia, the Nordic countries (Sweden, Norway, Denmark and Finland), the USA, the United Kingdom and Latin America (Brazil and Argentina). Our primary focus is on empirically studying the relationship between the increasingly complex diversities created by growing non-religion populations and institutions, and on building an evidence base from which to identify models for living well together in complex, diverse and inclusive societies. We have deliberately avoided investing time in protracted debates about the definition of non-religion and we have taken an oblique approach to measuring non-religion. By this we mean that rather than focusing on direct measures of religion or non-religion, we have developed a number of projects that examine everyday lives to understand the social construction of (non-)religion. In our fieldwork involving surveys and interviews, we avoid asking people specifically about religion or non-religion until the last possible moment, and we focus on what matters to people as a way to explore what they do rather than what they believe. One of these projects is the Community Gardens project.

We (the research team) have designed the Community Gardens project to explore the communities, practices, relationships and ethics that form in community gardens.^8^ We are interested in individuals as well as group practices and the broader notion of moral ecologies. Community gardens are spaces where relationships of interdependence develop among humans and between human and non-human beings. These relationships are diverse and entail cooperation in order to support the flourishing of the garden. Otherwise stated, the success of the garden is built on a plurality of moral relationships that unfold among humans and other-than-humans in the everyday (Ezzy, 2013). The research team began collecting data in fall 2021. We conducted one-on-one semi-structured interviews with community gardeners, both religious and non-religious, located in Argentina, Australia, Brazil, Canada, Finland, Norway, the United Kingdom and the USA. A total of 47 community gardens are represented in the project. The interviews primarily took place via Zoom but the researchers conducted interviews and visited gardens in person where possible. One focus group interview with refugees at a Canadian community garden also took place with the assistance of a translator. The purpose of this article is not to report in detail on the results of the project, but rather to think about what Piette’s approach offers in terms of methodological and conceptual advantages and inspiration.

We have resisted predefining non-religion, a stance that Piette might approve of, so that we can pay greater attention to social actors and their narratives of gardening in a community as they conceptualize it. Understandings of ‘community’ in community gardening can be limited to human members or expanded to include both human and non-human beings. Rob Moquin et al. (2016: 98) found that their community garden participants ‘recognized that community garden relationships extend beyond the social, and include complex ecological relationships with the plants, animals, and environmental systems that make up, and contribute to, their garden communities’. Beaman (2020: 256) explains in her research on sea turtle rescue volunteers that the line between human and non-human is ‘porous’ in that the volunteers perceive community as including the sea turtles. Notions of community can also be exclusionary. We recognize that community gardens are not free of problems, such as social or economic barriers to participation, or feelings of exclusion that inhibit building an inclusive sense of community (Neo and Chua, 2017). Exclusion can also be faced by the non-human beings for which the garden is home (Ginn, 2013). We thus pay attention to notions of community as depicted by our participants, both inclusive or otherwise, and how these may link to the ways garden members shape the moral ecology of the garden through practice.

The Community Gardens project uses a lived (non-)religion approach by focusing on how individuals experience and make sense of their practices and relationships with others in the garden. Piette also turns his attention to individuals by closely following the activities of one priest in *La Religion de près.* Piette does not isolate the priest as a social actor, however, choosing instead to concentrate on the priest’s interactions with his parishioners and the dialogue that emerges among them. Therefore, although Piette narrows his field of vision to a specific individual instead of a range of social actors,^9^ he situates his work in a relational or collective setting. Scholars of lived religion often treat religion as being social or relational despite focusing on individuals, albeit perhaps not to the extent of Piette’s sustained focus on the priest. As McGuire (2008: 12) states: ‘Although lived religion pertains to the individual, it is not merely subjective. Rather, people construct their religious worlds together, often sharing vivid experiences of that intersubjective reality’. Based on her research on Marion statue devotion, Amy Whitehead (2020) argues that lived religion can include relationships between humans and non-human persons such as religious statues. Our project approaches relationships as occurring between human and non-human beings within the collective space of community gardens, but we view these as being shaped by and understood from religious *and* non-religious perspectives.

The Community Gardens project does not follow an implicit religion path: gardening is not ‘like’ religion. Instead, it opens a beginning place for better understanding the production of non-religion in everyday life. Anna Sofia Salonen takes a similar approach in her research on eating animals:

Despite the growing interest in research on nonreligion, the way nonreligious identities are lived in the context of everyday life is still understudied. One relevant site for such research is the ﬁeld in which people express environmental concerns and engage in relationships with nonhuman animals and nature. (Salonen, 2019: 9)

The Community Gardens project seeks a deeper understanding of the complex and contradictory ways that social actors perceive and relate to the human and more-than-human world. This does not necessarily entail environmental concern that extends beyond the garden, such as climate change, but such concern can arise during the interviews. One non-religious garden coordinator, for instance, explained that ‘We’re just destroying [the world] every single day’, and her position at the community garden made a positive contribution by helping others see the connections they have with nature. This ‘connection’ was not called ‘religious’ or ‘spiritual’ by our participant, and we accordingly do not categorize it as such, following Piette’s cautious approach towards the use of sociological categories.

In terms of methodology, Piette’s ‘still life’ approach inspires us to take up a ‘slow sociology’ that focuses attention on routine, the detail of what people grow in their gardens, how they grow it, and so on.^10^ The questions we ask focus on the sights, smells and sounds of the gardens. We encourage the gardeners we interview to paint a visual picture of the garden for us. This is sometimes, admittedly, a bit of a tedious exercise. The participants count the beds in the gardens, hum and haw over measurements, and double back to self-correct. But if we take Piette seriously, such pixelated descriptions are part of the everyday that matters to our participants. We are tempering our desire to leap to ‘the point’ and to analysis. The questions also prompt the participants to describe their experiences and interactions with the garden and other garden members. This includes how they work the soil, share seeds with each other and engage in conversation while gardening. Sometimes language barriers prevent exchanges in conventional ways, and so we are also interested in the multiple ways in which the gardeners participate, including through gesture (offering food, tools, and so on).

As important as the detail of everyday life is, it is equally important not to rush into engaging in ‘higher-order’ theorizing. This is a difficult stance to take when one is trained to analyse and make sense of ‘data’. While we agree with Beckford (2017) that this is important and necessary, we are here arguing for a more careful, sustained study of the garden experience as one possible way to access a better understanding of lived (non-)religion. In so doing, we investigate the complexity of non-religion as revealed through our data, which, as Christopher Collins and Carrie Stockton (2018: 9) indicate, risks being limited by ‘an overreliance on theory’. This is particularly important when researching lived non-religion, for which more research needs to be done. It remains a relatively underdeveloped area of study, especially in relation to research on moral relationships with non-human animals or nature. The inclination to overanalyse data can lead to the territory of ‘religious sociology’ (Altglas and Wood, 2018), in which expansive definitions of religion are used to demonstrate that religion permeates social life. Any reference to the spiritual, transcendence or sacredness in this approach (for instance, ‘nature is sacred’) is brought under the umbrella of ‘religion’. A biased inclination towards non-religion can likewise erase aspects of religion (translated as ‘they are really atheists and so these religious engagements are meaningless’). Yet there are spaces in everyday life where religion and non-religion can manifest simultaneously or in contradictory ways.

We are inspired by Piette’s work to give greater attention to the messy contours of everyday life, specifically the contradictions embedded therein. Providing an opportunity for these contradictions to manifest is important in social science research, yet it is very often foreclosed by the imposition of categories that force narrative into pre-existing schema. This is partly a consequence of language and communication. Piette offers us an enhanced awareness of the implications of imposing categories. In our interviews with gardeners, contradictions abound. For instance, one interviewee, who self-identifies as an atheist, is good friends with a Baptist pastor and regularly participates in church activities that involve hikes and outdoor activities. Another, who also self-describes as non-religious, has connected a refugee to church resources as he feels these provide the best community-settlement support. He participates in church activities from time to time because he feels a sense of responsibility for the person, and keeping involved in the church is one way to keep tabs on their settlement process without being too intrusive in the person’s life. Piette erects an interpretive wall around such data, preferring to let voices speak for themselves. These examples demonstrate the paradoxes of non-religion that risk being overlooked if space for nuance and contradiction is not prioritized in our exploration of this social phenomenon.

Piette also offers an approach for capturing contradictions or disagreements among social actors. As previously mentioned, this is seen in *La Religion de près* when parish members disagree over what constitutes a proper religious burial. Other examples include debates over the proper interpretation of scripture and representations of Mary (Piette, 1999: 198–201). In the Community Gardens project, we seek to understand various (non-)religious ways of relating to the non-human world, some of which are complimentary and others in conflict. For instance, one interviewee, who describes himself as ‘very spiritual’, works to establish a reciprocal relationship in his community garden. This entails giving back to the garden and non-human animals that live there, such as by leaving weeds to grow that other animals or insects find valuable. Difficulty arises in the garden when other members do not share his outlook. In one instance, some potato bugs were found on a couple of potato plants, and some garden volunteers responded by squishing them all. The interviewee describes this as ‘a bit of a conflict’ because he would have preferred ‘to let things run their course and find balance on their own over time’. The community garden is a shared space where tensions can arise from different outlooks over correct practice.

As seen with the potato bugs, a struggle can arise between human and non-human members in the community garden. Another participant, who is ‘not formally religious’ but sees ‘spirituality’ as being ‘linked to nature’, describes how raccoons ate the corn in her community garden plot prior to harvest:

The fact that the raccoons came in and ate the corn, it was a shame. But I think there’s a sense that we share the park and the environment with, um, other living things. Certainly there are snakes there and birds there . . . Even though you try to put up netting or you try to ward them off, you can’t. It just, yeah, so. Take it with a grain of salt.

Encounters between gardeners and small animals or insects was a topic of discussion in many interviews. Another participant, who does not identify as religious and has “no belief”, described the “little orange beetles” that feed on the asparagus he grows. When he starts to harvest it, he leaves some asparagus to “give energy back to the roots”, and later in the year, he enjoys watching the interaction between the colourful beetles and the plants on which they run around. The casual acceptance expressed in these accounts differ from those of some other gardeners, who described groundhogs, moths and bugs as ‘pests’ or ‘problems’ to be solved.

A close focus on everyday engagements among social actors characterizes much of Piette’s work and, in *La Religion de près*, he contextualizes these engagements in the wider life of the parish. We similarly move beyond the individual to account for the lived relationships that take place in the broader context of the community garden. In particular, we direct our attention to the ways in which members navigate and interpret interactions that take place in the garden, ranging from squishing potato bugs or deterring pests, to letting things “run their course”. These encounters were not linked by our participants to their religious or non-religious identities. Not killing snakes or slugs, for instance, was not framed as a spiritual imperative. This is not to say that (non-)religion is sociologically irrelevant. Analyzing these findings from a sociological perspective would involve factoring in the socially constructed relationships or hierarchies between humans and their non-human ‘others’, which may reflect or challenge traditionally religious notions such as ‘stewardship’ (Beaman and Strumos 2023). Rather, following the micro-level approach of Piette, to categorise these relationships as either ‘religious’ or ‘nonreligious’ would impose scholarly assumptions that are not reflective of participants’ own voices. Inspired by this approach, while maintaining an entry point of lived (non-)religion, we seek to identify how religion and non-religion are at play in our data without magnifying their presence.

## Conclusions

It is to these sorts of mundane ‘happenings’ that Piette turns in his study of religion, which blurs the boundaries of religion and non-religion, de-emphasizing its specialness and emphasizing its social construction in the activities of day-to-day life. Piette does not use the term ‘non-religion’, but his approach is focused on the making of religion as distinct from what is not religion. His concern is less with revealing the coherence or functionality of social processes or typical situations than with showing the indeterminacy of acts and gestures that might develop in paradoxical or unexpected ways. Studying religion as Piette suggests might easily be misinterpreted as a will to religion (Beaman, 2013), which sees everything imbued with religious meaning and expands the boundaries of religion’s reach. We think that Piette’s approach does the opposite. It contextualizes religion in the ordinariness of social life with no claim to a unique place in a hierarchy of human activity. Beckford summarizes this in his assessment of Piette’s approach:

Piette shows no intention of testing the sociological thesis that religion somehow spills over into areas such as politics or sport in conditions of modernity, but he focuses on the ways in which conventional religious ideas interact with non-religious ideas to form hybrids. This is a further indication of his desire to deflate the high theoretical ambitions of sociologists of religion by revealing the interweaving of religion and non-religion in perfectly mundane circumstances (Piette 2003, 101). (Beckford, 2017: 186)

Although the focus on the mundane resonates with the lived religion ‘turn’, Piette’s approach is less about discovering the non-institutional and creative ways in which people are religious and more about paying attention to the details of ‘religion in the making’ in everyday life. This might seem like a minor difference, but his emphasis on ‘religion in the making’ is important as we think about how people live and make non-religion. His focus on contradiction poses a challenge to uniform narratives and ideal types that gloss over difference, complexity and the motion that is inherent in social life.

Piette’s work has limitations for the study of everyday religion from a sociological perspective. Beckford (2017) highlights that social life is influenced by structural forces, so a comprehensive understanding of religion calls for a consideration of power, histories and frames of meaning alongside the ‘minor mode’ of religion that characterizes Piette (1999, 2015). Altglas and Wood (2018: 6) argue that sociologists of religion should move beyond ‘the narrow description of religion for itself’ and treat religion as ‘a social phenomenon that needs to be studied in conjunction with other social trends and facts – the family, authority, work, ethnicity, health, and so forth’. Wood offers a critical analysis of sociological research on congregations and highlights the need for attention to power in this area:

What do congregational studies tell us about the sociology of religion today? In general, they are symptomatic of weaknesses that prevail in this field of study. Phenomena are increasingly presented as socially decontextualised: they are lifted out of the social contexts necessary to make sense of them but which researchers dismiss because they involve issues of social power or decision-making. Linked to this social decontextualisation is the way phenomena are presented – in a manner that strips them of their practical and interactional dimensions. There is frequently little interest in what people are doing and with whom, even though most congregational studies make use of participant observational research. (Wood, 2018: 116)

Piette partially addresses this issue in *La Religion de près* with his rigorous inspection of the ‘practical and interactional dimensions’ of parish life, but he does not factor in ‘issues of social power’ through subsequent analysis. Even if we disagree on the need for an interpretive move by the social scientist,^11^ Piette’s careful attention to hesitations, doubts, contradictions and paradoxes is an important reminder of the social in social science. We think this is especially important in the study of non-religion as we enter this relatively new sociological territory.

We have drawn inspiration from Piette’s work, particularly *La Religion de près*, as we think about how to study non-religion, which poses unique definitional, conceptual and mapping challenges. However, we have not done the deep ethnographic and descriptive work that Piette does, in large measure because the COVID-19 pandemic dramatically shifted how we conducted this research. Our initial vision of the research project, which involved participant observation in community gardens, was of necessity replaced by a more modest research strategy. We intended to be immersed in the growing, sharing and action that takes place in community gardens, but our attention has shifted to the moral ecologies and relationships that unfold in these spaces. The overall intention of our discussion here is not to report on our research findings. Instead, our aim has been to explore the challenges of researching lived (non-)religion in conversation with Piette. We have taken seriously his approach and see non-religion as something ‘in the making’, rendering it a complex phenomenon that warrants a careful and cautious social scientific approach.

## Notes

1. Piette (1999: 27) writes: ‘Croyance a des êtres surnaturels, rapport au sacre, univers de sens, ensemble de significations ultime, rapport explicite a une tradition . . . : les définitions se sont ainsi multipliées, le plus souvent centrées sur un seul trait juge spécifique du fait religieux’. (‘Belief in supernatural beings, relationship to the sacred, universe of meaning, set of ultimate meanings, explicit relationships to a tradition…: the definitions are thus multiplied, most often centered around a spectific feature of religious reality’.)

2. We will thus describe what people do when they deal with a religion, when they “do” religion, through confusion and the daily crumbling of encounters. We will dive into the heart of religious activity by following the actors as closely as possible, in their local interactions, the anecdotal details of verbal exchanges and the most insignificant gestures. Translations by Mathilde Vanasse-Pelletier.

3. American sociologists working with the rational-choice theory of religion have argued that people are ‘religious consumers’ who decide what groups or movements to join based on the benefits or rewards they offer, including social capital and eternal life (Iannaccone, 1992; Stark and Bainbridge, 1987). Davie (2007: 86) indicates that rational-choice theory resonates more with the American context than that of Europe, where people ‘regard their churches as public utilities rather than competing firms’.

4. Piette (1999 : 31) asks: ‘Pourquoi éliminer les circonstances pratiques et les faits ordinaires alors que ce sont eux qui font, qui construisent les activités dont les effets ou les causes servent de support aux interprétations macrosociologiques? Même si l’on sait que la religion n’est pas une essence, ni même un objet naturel, on continue à se braquer sur elle-même, au lieu de chercher à décrire les pratiques dont elle n’est que la projection’. (‘Why eliminate practical circumstances and ordinary facts when they are the ones doing, constructing the activities whose effects or causes help support microsociological interpretations? Even though we know religion isn’t an essence, or even a natural object, we contiue to focus on it, instead of aiming to describe the practices which religion is a reflection of’.)

5. Ammerman (2016: 91) argues: ‘Some practices are deeply embedded in religious tradition, while others engage sacred realities with words and forms that borrow widely. . . . If we grant that even the most apparently staid and hierarchical religious tradition is nevertheless enacted – practiced – then there is room to study the way elite religious discourses are lived, using the methodological and theoretical tools lived religion research has taught us’. Piette can be said to demonstrate in *La Religion de près* the benefits of using ethnography to capture lived religious experience in a ‘staid and hierarchical’ setting.

6. Piette (1999: 78) explains: ‘C’est lui aussi qui fait agir les hommes et les femmes que nous avons rencontres, qui les pousse a se reunir pour réorganiser la paroisse et le faire advenir dans une célébration dominicale, a le prier, a accomplir des actions humanitaires, a aller chercher des fleurs, des hosties, du vin’. (‘It is alo he who makes the men and women we have met with act, who pushes them to gather to reorganize the parish and bring him forth during a Sunday celebration, to pray to him, to take part in humanitarian action, to go and get flowers, wafers, and wine’.)

7. Beyer (2015: 137) distinguishes between ‘lived atheism’, which denotes people who ‘constitute atheism through their personal activity and consciousness’, and ‘systematic atheism’, which is ‘how the activity of people together contributes to the construction of atheism as movement, as organization, and as institution, that is, as social system’.

8. The Community Gardens research project is led by Lori G Beaman and Douglas Ezzy. At the time of writing the working group consisted of Ryan T Cragun (Co-Investigator); Mary Jo Neitz (Advisor); Rebecca Banham (Postdoctoral Fellow); Anna Sofia Salonen (Postdoctoral Fellow); Mathilde Vanasse-Pelletier (Postdoctoral fellow); Alexander Burton (Research Assistant [RA]); Giovanna Paccillo (RA); Iriana Sartor (RA); Jonathan Abernethy-Barkley (RA); Kendall Stephenson (RA); Lauren Strumos (RA); and Marziyeh Riazi (RA).

9. Piette (1999: 185) writes: ‘Ainsi, cette série ethnographique rétrécit notre champ de vision puisqu’il ne s’agit plus de suivre différents acteurs éparpilles dans des groupe-ments paroissiaux différents, ni les mêmes acteurs en des réunions différentes dans un même ensemble paroissial; mais, en même temps, elle accentue la proximité de l’objet observe puisqu’il s’agit de suivre sans interruption une seule personne dans toutes ses activités’. (‘Thus, this ethnographic series shrinks our field of vision since it’s not about following different actors scattered into different parishes, or even the same actors through different meetings inside of the same parish anymore, but, at the same time, it emphasizes the proximity of the object we are looking at, since it’s about following one person throughout all of their activities’.)

10. The term ‘slow sociology’ is used by Garey et al. (2014) to describe research that unfolds over a relatively long period of time and challenges the ‘quantity over quality’ trend found in much of academia. We use the term to signify a particular approach that aligns with Piette’s micro-examination of social processes.

11. And we are not so sure that we do disagree. In his later work, Piette describes his ‘existential anthropology’ as producing bridge descriptions to other descriptions on different scales. He notes sociology as being ‘larger scale’, which is the case in some instances, but underestimates sociology’s phenomenological strand (Piette, 2015: 182).
